# Responses of species abundance distribution patterns to spatial scaling in subtropical secondary forests

**DOI:** 10.1002/ece3.5122

**Published:** 2019-04-01

**Authors:** Anchi Wu, Xiangwen Deng, Honglin He, Xiaoli Ren, Yiran Jing, Wenhua Xiang, Shuai Ouyang, Wende Yan, Xi Fang

**Affiliations:** ^1^ Faculty of Life Science and Technology Central South University of Forestry and Technology Changsha China; ^2^ National Engineering Laboratory for Applied Technology of Forestry＆Ecology in South China Changsha China; ^3^ Huitong National Station for Scientific Observation and Research of Chinese Fir Plantation Ecosystems in Hunan Province Huitong China; ^4^ Key Laboratory of Ecosystem Network Observation and Modeling, Institution of Geographic Sciences and Natural Resources Research Chinese Academy of Science Beijing China; ^5^ Graduate University of Chinese Academy of Sciences Beijing China; ^6^ Chinese Research Academy of Environmental Sciences Beijing China

**Keywords:** community structure, neutral theory model, niche model, secondary forests, spatial scaling, species abundance distributions

## Abstract

To quantify and assess the processes underlying community assembly and driving tree species abundance distributions(SADs) with spatial scale variation in two typical subtropical secondary forests in Dashanchong state‐owned forest farm, two 1‐ha permanent study plots (100‐m × 100‐m) were established. We selected four diversity indices including species richness, Shannon–Wiener, Simpson and Pielou, and relative importance values to quantify community assembly and biodiversity. Empirical cumulative distribution and species accumulation curves were utilized to describe the SADs of two forests communities trees. Three types of models, including statistic model (lognormal and logseries model), niche model (broken‐stick, niche preemption, and Zipf‐Mandelbrodt model), and neutral theory model, were estimated by the fitted SADs. Simulation effects were tested by Akaike's information criterion (*AIC*) and Kolmogorov–Smirnov test. Results found that the Fagaceae and Anacardiaceae families were their respective dominance family in the evergreen broad‐leaved and deciduous mixed communities. According to original data and random sampling predictions, the SADs were hump‐shaped for intermediate abundance classes, peaking between 8 and 32 in the evergreen broad‐leaved community, but this maximum increased with size of total sampled area size in the deciduous mixed community. All niche models could only explain SADs patterns at smaller spatial scales. However, both the neutral theory and purely statistical models were suitable for explaining the SADs for secondary forest communities when the sampling plot exceeded 40 m. The results showed the SADs indicated a clear directional trend toward convergence and similar predominating ecological processes in two typical subtropical secondary forests. The neutral process gradually replaced the niche process in importance and become the main mechanism for determining SADs of forest trees as the sampling scale expanded. Thus, we can preliminarily conclude that neutral processes had a major effect on biodiversity patterns in these two subtropical secondary forests but exclude possible contributions of other processes.

## 1 INTRODUCTION

Against the background of global change, concerning about rapid biodiversity loss has intensified the need to better understand community structure, and investigating the processes that determine the relative abundance of species in a community has long been a central task in ecology (Borda‐De‐Água, Borges, Hubbell, & Pereira, 2012; Magurran & Henderson, 2003; Rosenzweig, 1995). The species abundance distributions (SADs), which describes the abundances of all species within a community (Ulrich, Ollik, & Ugland, 2010), have been used extensively to examine the influence of niche differentiation, dispersal, density dependence, speciation, and extinction on the structure and dynamics of ecological communities (Green & Plotkin, 2007), and have played a major role in the development of prominent theories of biodiversity and biogeography (McGill et al., 2007).

Since Motomura (1932) first attempted to reveal the underlying mechanism of species abundance curves, various theoretical models have emerged, whereas the recent review by McGill et al. (2007) listed 27 different species abundance models. The two earliest and most widely used models have been the logseries (Fisher, 1943) and the lognormal (Preston, 1948), while multiple models in an abundance distribution were later tested using a Poisson lognormal model (Bulmer, 1974; Matthews, Borges, & Whittaker, 2014). Meanwhile, many potential mechanisms driving the aforementioned multimodality have been proposed, including elaborate mechanistic models of niche division (Tokeshi, 1999), ecological drift (Hubbell, 2001), or spatial distribution (Harte, Kinzig, & Green, 1999). Recent studies on SADs mainly focus on fitting the observed data using the above models, and then inferring the mechanism maintaining species diversity based on their respective fitting effects (Alonso, Ostling, & Etienne, 2008; McGill et al., 2007; McGill, 2011; White, Thibault, & Xiao, 2012). However, Niche and neutral theories have long been controversial in explaining different processes contributing to the maintenance of species diversity. Niche theory proposes that, in a community at equilibrium, each species must occupy a different niche, emphasizing the importance of environmental filtering or species interactions in determining species composition (Gilbert & Lechowicz, 2004); neutral theory contrarily, assumes spatial processes alone determine species composition (Chisholm & Lichstein, 2009; Hubbell, 2005), that requires understanding the mechanisms through which species are able to coexist and provides a theoretical framework for exploring community structure at the individual level (Chave, 2004; Pimm, Russell, Gittleman, & Brooks, 1995).

Subtropical broad‐leaved forest, a major biome worldwide that covers an extensive area and supports high biodiversity in eastern Asia, has a community structure and species composition quite different from those of tropical and temperate forests (Corlett & Hughes, 2015; Jin, Qian, & Yu, 2015). China has sustained a high human population density for much of its history, and severe human disturbances have left few pristine forests in this country, such that most existing forests are in early‐ or mid‐successional phases (Feng et al., 2014). Elucidating the relative importance of processes that determine community assembly is particularly urgent in human‐impacted landscapes, where recovery from disturbance has critical implications for conserving unique biodiversity and for rehabilitating ecosystem services (Chazdon, 2008; Harvey et al., 2008). Some researchers claim that human‐impacted communities have been so disrupted that species composition will never return to its original state, and secondary forests could serve as biodiversity reservoirs for mature forest species (Aide & Grau, 2004; Brook, Bradshaw, Lian, & Sodhi, 2006; Norden, Chazdon, Chao, Jiang, & Vílchez‐Alvarado, 2009).

Used abundance data from two 1‐ha plots in China's subtropical secondary forests, we explored how spatial scaling influenced maintenance mechanisms of two typical community. Six models were fitted and analyzed for species abundance patterns at different scales, which every model represented the fundamental processes determined the behavior of the communities. Hence, the objectives of this study were threefold: (i) to compare the community structure and composition change between an evergreen broad‐leaved and deciduous mixed forest; (ii) to quantify their SADs and identify an optimal model at different spatial scales; and (iii) to explain the processes underpinning the SAD patterns and mechanisms of community assembly in subtropical secondary forests. Overall, our study has established a framework to understand mechanism and ecological process of abundance patterns formation with spatial scaling, aiming to improve the conservation and management of subtropical forests.

## 2 METHODS

### 2.1 Study site

Dashanchong Provincial Nature Reserve (28°23′58″–28°24′58″N, 113°17′46″–113°19′08″E) is located about 45 km away of Changsha City, Hunan Province, in central southern China. The region experiences a monsoonal climate characteristic of mid‐subtropical regions. Altitude ranges from 55 to 218 m above sea level and slopes range from 7.5° to 78.2°. The annual precipitation ranges from 936.4 to 1954.2 mm, averaging 1,416.4 mm, with a yearly frost‐free period lasting from 278 to 300 days, and the annual mean temperature and relative humidity level are 16.8°C and 87%, respectively. The soil type is red soil, developed from slate and shale rock, with pH 4.0–4.9. The primary vegetation type is subtropical evergreen broad‐leaved forest, but this was largely destroyed by human disturbances. However, after a long period of forest succession, about 60 years, much natural secondary vegetation now exists and is preserved. The canopy of two forest types was fully closed.

### 2.2 Plant census

In 2014 and 2015, we established two 1‐ha permanent study plots in the best‐preserved forest areas of the study region, which included, respectively, *Lithocarpus glaber–Cyclobalanopsis glauca *evergreen broad‐leaved forest (LC) and *Choerospondias axillaries* deciduous mixed forest (CA), both being typical vegetation communities of Chinese subtropical forest types. Each plot measured 100 m by 100 m, with its axis running from southeast to southwest, and was divided into 100 subplots (10 × 10 m). Conducted a plant census in every subplot, all tree stems with diameter at breast height (1.3m, DBH) ≥1 cm were tagged, identified, and measured for their DBH, total height (H), and crown width. In addition, their species names, health status, and spatial coordinates were recorded. Using tape rulers, the spatial coordinates of each stem had two‐dimensional accuracy of  ±15 cm. Although most live individuals were identified to species, in those cases where plant identity was uncertain, voucher specimens were collected and labeled for subsequent identification at Flora of China (http://frps.eflora.cn).

### 2.3 Sampling methods

Within the 100 × 100 m plots, a point was randomly selected, which served as the starting point and was extended 10 m to the *x*‐ and *y*‐axis, respectively, to form a superimposed subplot of 10 × 10 m sampling scale for using in the simulation. Similarly, we obtained increasingly larger‐size subplots of 20 × 20, 40 × 40, 60 × 60, 80 × 80, and 100 × 100 (m × m) with the same starting point. The highest level of species was 1, the second level was 2, and so on. Furthermore, used plot‐level histograms to express the SADs of each community, all plot‐derived samples were plotted as histograms using a log_2_ binning method, whereby bin 1 equal the number of singleton species, bin 2 equal 4–7, and so on (Matthews et al., 2014).

### 2.4 Data analysis

The relative importance value (RIV) for each tree species in the two forest community types was calculated as the function of its average of relative density, relative basal area, and relative frequency on a percentage basis (Liu et al., 2014).To measure species diversity in each community, we used four indices: species richness (*S*), Shannon–Wiener (*H'*), Simpson(*D*), and Pielou (*E*) (Magurran, 1988); the specific calculation formulas were as follows: $H^{\prime }=-\sum P_{i}\text{In}P_{i}$; $D=1-\sum P_{i}^{2}$; $E=H^{\prime }/\text{In}S$. Where *S* is the number of species in the sample square, *P*_i _is the important value of the species i in the community.

The species accumulation curves (SAC) were used to compare the diversity properties of community samples (Colwell et al., 2012). Instead of the commonly used probability density function, the empirical cumulative distribution function (ECDF) may be plotted, which was useful for highlighting differences in SADs between communities (Matthews & Whittaker, 2015).

Niche and neutral theories emphasize different processes contributing to the maintenance of species diversity (Holyoak, Loreau, & Strong, 2006; Rominger, Miller, & Collins, 2009). To evaluate the relative importance of niche‐based deterministic processes and neutrality‐based stochastic processes in community structure, three types models were used to explain and quantify the pattern and process of species abundance distributions. To determine whether the SADs of the various samples exhibited multiple modes, the logseries, lognormal, broken‐stick, niche preemption, Zipf‐Mandelbrodt, and neutral theory models were fitted to the SADs data of each forest type (Fisher, 1943; Hubbell, 2001; MacArthur, 1957; McGill et al., 2007; Preston, 1948). The first two models were purely statistical, the middle three were niche‐based, and the last one followed neutral theory. To test their goodness‐of‐fit, we used the Kolmogorov–Smirnov test (*K‐S *test) (Sekhon, 2011) and Akaike's information criterion (*AIC*) (Burnhan & Anderson, 2002). Generally, the smaller the *AIC* value, the more robust the fit. The *K‐S* test was used to fit the difference between the results and the actual observed values. When *p < *0.05, the model was rejected. All analyses were conducted using R statistical software (R Development Core Team, 2017) with the packages vegan, fossil, and sads.

## 3 RESULTS

### 3.1 Community structure and composition

**Figure 1:**
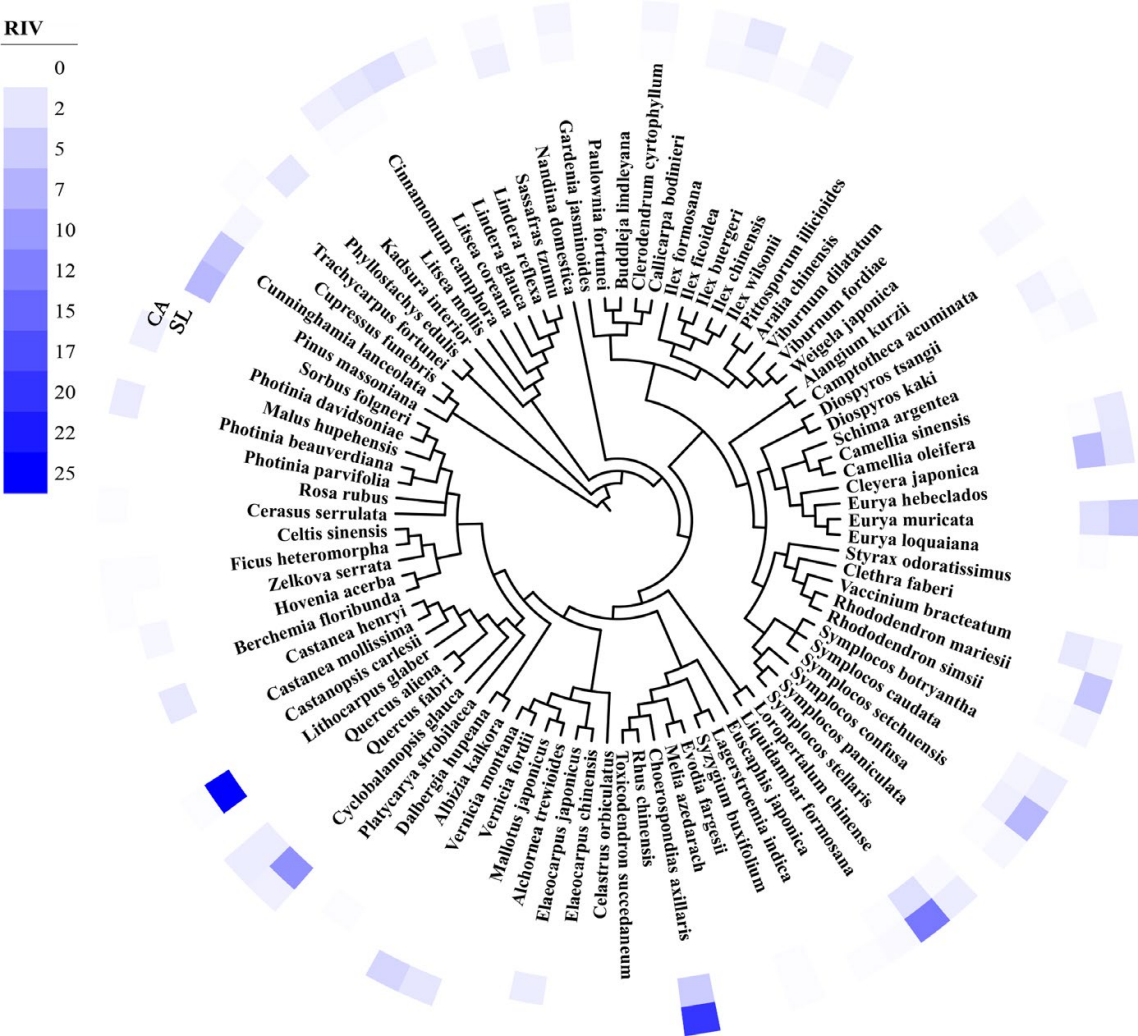
A community phylogeny in Dashanchong and the relative importance value (RIV) in the *L. glaber–C. glauca *evergreen broad‐leaved forest (LC) and *C. axillaries* deciduous mixed forest (CA) communities

We surveyed 4,805 woody plants individuals belonging to 76 species, 38 families, and 55 genera in the LC community, for which the RIV of *L. glaber* and *C. glauca* were 25.30 and 15.03, respectively. The Fagaceae reaching the RIV of 35.94 was the most dominant family. In terms of their composition, evergreen and deciduous tree species occurred in similar proportions (i.e., evergreen broad‐leaved = 50.68% vs. deciduous = 49.32%). However, the woody plants consisted of 5,361 individuals representing 70 species, 28 families, and 44 genera in the CA community, for which evergreen and deciduous tree species proportions constituting 38.57% and 61.43%, respectively. The dominant species which was *C. axillaries* belonging to Anacardiaceae had the highest RIV (20.02) in this community (Figure 1).

**Figure 2:**
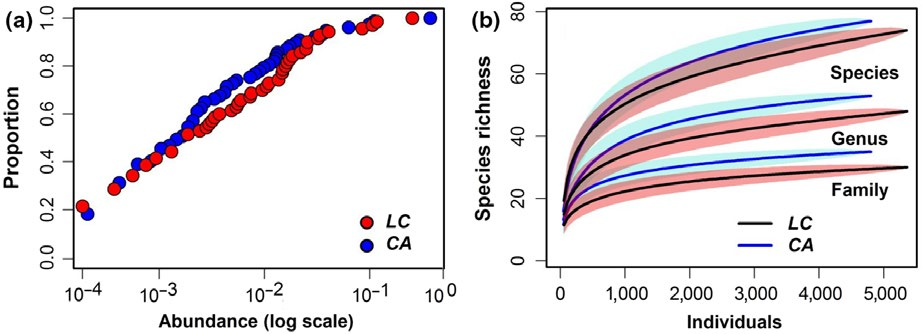
Different ways to plot the SADs in the *L. glaber–C*. *glauca *evergreen broad‐leaved forest (LC) and *C. axillaries* deciduous mixed forest (CA) communities. (a) Trees’ hypothetical SADs plotted using the empirical cumulative distribution function (ECDF). (b) Comparison of the curves for individuals with 95% unconditional intervals

The ECDF was similar between the LC and CA plots, but the proportion of species individual proportion in CA was significantly higher than that in LC in these secondary forests communities (Figure 2a). Furthermore, the SAC revealed the species richness of LC consistently exceeded that of the CA as number of sampled individuals increased, which the similar reasoning was applicable to patterns of species‐to‐family (Figure 2b).

### 3.2 Variation of species abundance distribution trends

**Figure 3:**
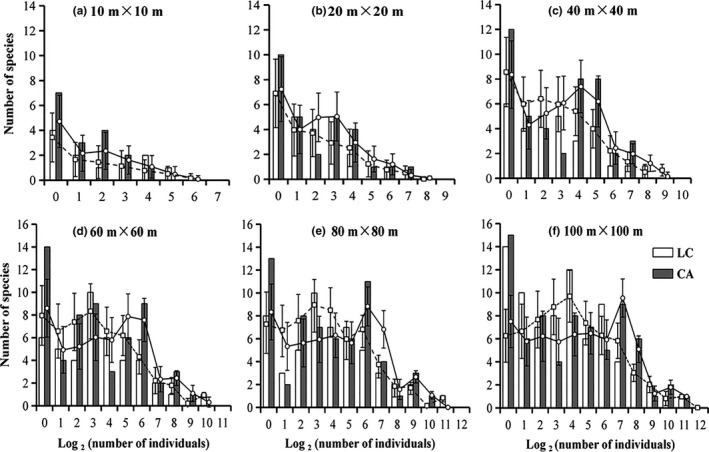
Species abundance diagrams at different scales in the *L. glaber–C*. *glauca *evergreen broad‐leaved forest (LC) and *C. axillaries* deciduous mixed forest (CA) communities. Error bars represent standard deviations obtained with 1,000 randomly repetitions. (a) to (f) represent the increasing spatial scales of plant sampling (m × m)

Individuals of common species occupied a large proportion of the community trait space under different sampling scales. Rare species richness also showed significant differences at different spatial scales, and it was higher in deciduous mixed forest community. Random sampling results revealed that SADs changed their shape as the sample size changed. With examination of sampled distributions, results demonstrated that the SAD for areas between 0.1 and 1.0 ha changed from a left‐skewed monotonically decreasing distribution to one with a maximum for intermediate abundance classes. This maximum steadily appeared between 8 and 32 in the LC community, but proved fluctuant change with the sampled area size in the CA community (Figure 3).

### 3.3 Model comparisons and selection

We estimated SADs for woody plants by fitting neutral, lognormal, logseries, broken‐stick, niche preemption, and Zipf‐Mandelbrodt models at varying sampling scales. At smaller scales (10 × 10 m and 20 × 20 m), all six models passed the *K‐S* test and were within an acceptable range (*p* > 0.05). In particular, the neutral, purely statistical, and broken‐stick models all fitted well to the data, and *AIC* values kept small difference among models. When the sampling scale exceeded 40 × 40 m, three niche models were fitted poorly and were even rejected (*p < *0.05). Both the neutral and purely statistical models fitted better, with the logseries model the best overall (Table 1).

**Table 1:** Goodness‐of‐fit tests of six models for the species abundance distributions of the *L. glaber–C*. *glauca *evergreen broad‐leaved forest (LC) and *C. axillaries* deciduous mixed forest (CA) communities

Forest type	Scale (m)	NTM		LNM		LSM		BSM		NPM		ZMM	
		*AIC*	*D*	*AIC*	*D*	*AIC*	*D*	*AIC*	*D*	*AIC*	*D*	*AIC*	*D*
LC	10 × 10	79.0	0.07	85.3	0.27	79.1	0.07	87.8	0.27	406.0	0.33	406.2	0.40
	20 × 20	125.5	0.13	124.8	0.17	125.1	0.13	125.7	0.26	135.9	0.26	713.2	0.25
	40 × 40	239.6	0.14	238.0	0.11	238.8	0.11	266.7	0.29	2,790.5	0.31*	2,567.0	0.14
	60 × 60	336.2	0.14	336.2	0.09	334.9	0.12	392.4	0.37**	6,769.9	0.40**	6,219.1	0.26*
	80 × 80	458.3	0.09	461.4	0.08	457.3	0.09	540.0	0.40***	15,142.2	0.41***	14,130.4	0.28*
	100 × 100	644.4	0.13	655.0	0.12	642.8	0.12	805.3	0.47***	26,931.7	0.48***	24,932.3	0.39***
CA	10 × 10	55.9	0.06	58.5	0.25	56.2	0.06	60.0	0.13	202.3	0.25	203.5	0.44
	20 × 20	178.2	0.19	184.8	0.22	178.6	0.19	180.4	0.16	1,232.8	0.25	1,259.5	0.41
	40 × 40	304.3	0.14	318.2	0.16	304.5	0.14	330.3	0.30*	4,161.4	0.18	4,222.3	0.48***
	60 × 60	456.0	0.10	471.4	0.12	455.9	0.10	532.2	0.41***	11,224.6	0.34**	11,245.8	0.44***
	80 × 80	536.5	0.09	549.8	0.11	536.1	0.06	630.2	0.37***	20,096.3	0.34***	19,619.1	0.44***
	100 × 100	644.4	0.07	664.7	0.13	644.1	0.07	771.2	0.40***	32,096.8	0.33***	31,717.0	0.46***

Note

*AIC*, Akaike's information criterion; *D*, K‐S test. NTM, LNM, LSM, BSM, NPM, and ZMM represent neutral theory, logseries, lognormal, broken‐stick, niche preemption, and Zipf‐Mandelbrodt model, respectively.

* *p < *0.05

** *p < *0.01

*** *p < *0.001.

**Figure 4:**
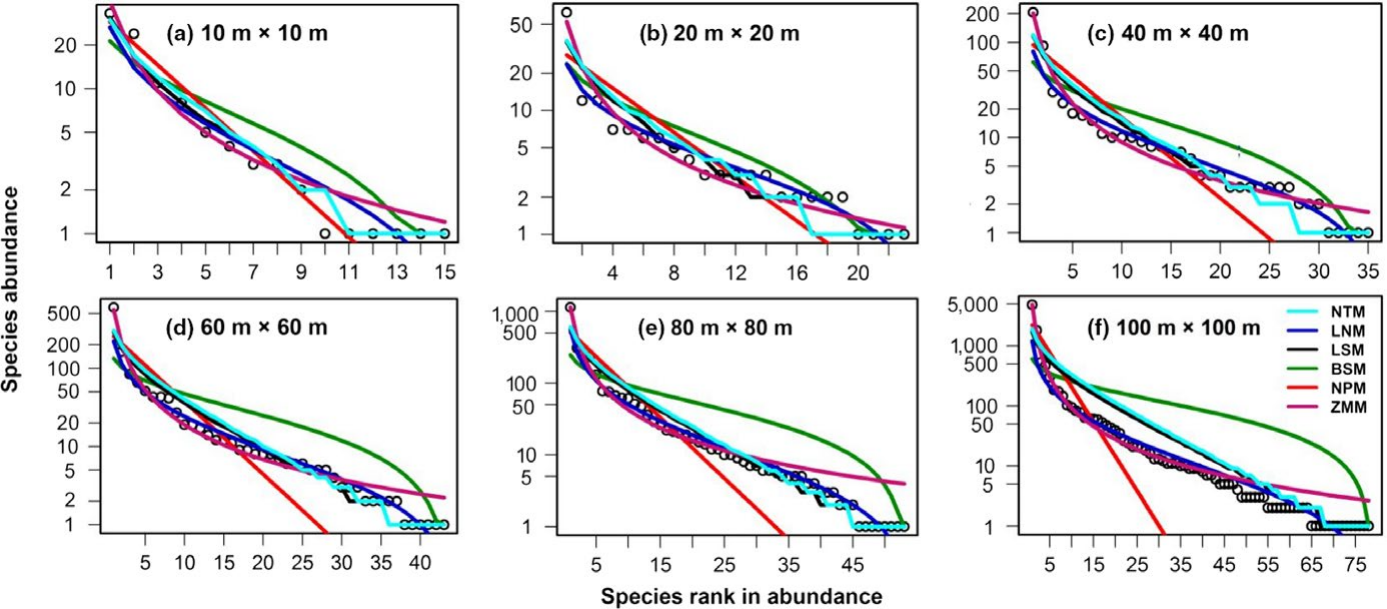
Species abundance distributions and model fitting for the *L. glaber–C. glauca *evergreen broad‐leaved forest community. (a) to (f) represent rank‐abundance plots and the fitting of six models, at increasing spatial scales of plant sampling (m × m). Observed values are shown as open circles. NTM, LNM, LSM, BSM, NPM, and ZMM represent neutral theory, logseries, lognormal, broken‐stick, niche preemption, and Zipf‐Mandelbrodt model, respectively

**Figure 5:**
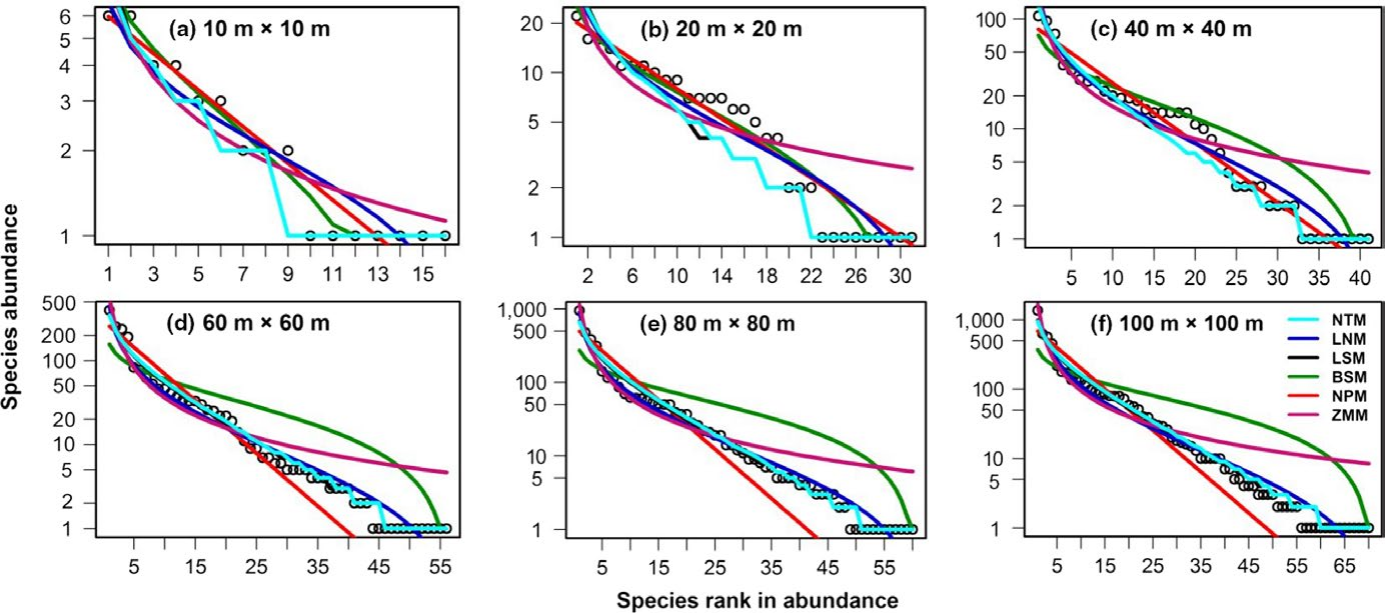
Species abundance distributions and model fitting for the *C.  axillaries* deciduous mixed forest community. (a) to (f) represent rank‐abundance plots and the fitting of six models at increasing spatial scales of plant sampling (m × m). Observed values are shown as open circles. NTM, LNM, LSM, BSM, NPM, and ZMM represent neutral theory, logseries, lognormal, broken‐stick, niche preemption, and Zipf‐Mandelbrodt model, respectively

Different models had different fitting effects on common, intermediate, and rare woody plant species across sampling scales. The neutral and logseries models, for example, provided a better fit for the SADs of these two typical secondary forest communities, which was consistent with actual observations. It is worth emphasizing that the above two models fitted poorly to the intermediate species and were obviously overestimated at the 100‐m × 100‐m sampling scale in the LC community (Figure 4f). Meanwhile, the lognormal model had a poor fit to rare species. Niche models fitted well at the smaller sampling scale; nonetheless, their fitting effects worsened as sampling scales was enlarged. However, the SADs’ prediction of the intermediate species and rare species by the broken‐stick model obviously exceeded their actual values, whereas the common species were considerably under‐predicted at a great sampling scale. The niche preemption model provided better fitting for the common species, but its predictions for intermediate and rare species were smaller than their actual values. The predictions made for rare species by the fitted Zipf‐Mandelbrot model were larger than their actual values (Figures 4 and 5).

## 4 DISCUSSION

The composition of these two typical secondary forest communities demonstrated obvious differences among their dominant species, proportion of evergreen and deciduous species, biodiversity and regeneration dynamics. The original empirical data and random sampling methods revealed this pattern played an important role in determining the species abundance distribution shape: monotonically decreasing or bimodal. Borda‐De‐Água et al. (2012) found that, for areas above 50 ha, the species abundance distribution had a bimodal shape with a local maximum occurring for the singleton classes, but this maximum increased with size of the sampled area, and when we moved from 12.5 to 50 ha, the number of singletons increased yet the abundance classes of species with 2 and 3 to 5 individuals decreased. However, as Figure 3 showed, the singletons increased while the abundance classes of species with 2 individuals decreased at different spatial scales. The shapes of the extrapolated SADs were compatible with theoretical predictions and empirical SADs reported by other researchers (e.g., Kůrka, Šizlinga, & Rosindellb, 2010; Borda‐De‐Água et al., 2012).

For the SADs of two different typical forest communities in China's subtropical secondary forests, the results indicated that a clear directional trend toward convergence and similar predominating ecological processes. There existed different ecological processes dominated different sampling scales in our simulations. Overall, a purely statistic model could describe the species abundance structure and its quantitative relationship, but these were better explained by the neutral and niche process at smaller sampling scales. The fitting effect of niche models clearly worsened as the sampling area enlarged and was eventually rejected. It has been found that the neutral process gradually replaced the niche process and became the major mechanism maintaining the SADs with the spatial scales increasing. Therefore, multiple models should be applied when we studied the SADs at different sampling scales. The SADs showed a clear directional trend toward convergence with mature forests at Tiantong (20 ha) and Gutianshan (24 ha) in a subtropical broad‐leaved forest (Cheng, Mi, Ma, & Zhang, 2001; Fang et al., 2016).

Furthermore, both model and analytical study have revealed that neutrality increased with species richness, species diversity, and speciation rate (Bar‐Massada, Kent, & Carmel, 2014; Chisholm & Pacala, 2010; Kadmon & Allouche, 2007). With the spatial scale gradually increased in subtropical secondary forests, the species migration rate decreased while their local transmission increased. When the species richness, Shannon–Wiener index and Simpson index increased, the Pielou index gradually decreased along the scaling gradient (Table 2). Meanwhile, environmental heterogeneity affects many ecological patterns and processes—such as species abundance, species coexistence, species diversity, and community composition, and movement and dispersal of organisms (Snyder & Chesson, 2004); therefore, it may have an important influence on the final location of communities along the niche–neutrality continuum. The location of a community depends not only on its intrinsic characteristics but also on the interaction of the niche and neutral processes that drive community dynamics. Fisher and Mehta (2014) argued that niche and neutral processes were not the opposite in the community construction. While communities may be more or less neutral, no community is truly neutral or fully niche‐based; thus, the ends of the niche–neutrality continuum only exist in theory (Adler, Hillerislambers, & Levine, 2007; Purves & Pacala, 2005). Hence, we speculate that, in determining community species composition, the corresponding effects of neutral processes may strengthen when the effects of niche processes decline.

**Table 2:** Predicted parameters of neutral theory and species diversity indices at different spatial scales for the *L. glaber–C*. *glauca *evergreen broad‐leaved forest (LC) and *C. axillaries* deciduous mixed forest (CA) communities

Scale (m)	LC							CA						
	N	S	H′	D	E	θ	m	N	S	H′	D	E	θ	m
10 × 10	49	15	1.77	0.67	0.65	7.26	0.167	39	16	2.27	0.85	0.82	8.79	0.372
20 × 20	150	23	2.29	0.78	0.73	6.69	0.526	198	30	3.05	0.92	0.90	10.60	0.136
40 × 40	479	33	2.35	0.79	0.67	7.65	0.442	680	41	2.97	0.92	0.81	10.41	0.016
60 × 60	1,330	43	2.26	0.93	0.60	7.51	0.022	1905	57	2.84	0.90	0.70	11.28	0.032
80 × 80	2,874	53	2.51	0.80	0.63	8.49	0.013	3,490	61	2.84	0.96	0.69	10.35	0.027
100 × 100	4,805	76	2.63	0.81	0.61	11.94	0.005	5,364	70	2.91	0.89	0.68	11.44	0.127

Note

N, individual number; *S*, species richness; *H'*, Shannon‐–Wiener index; *D*, Simpson index; *E*, Pielou index; *θ*, fundamental biodiversity number; *m*, immigration rate.

Since tropical forest communities may be more strongly structured by neutral processes than boreal forests, they may be located closer to the neutral end of the continuum axis (Chisholm & Paccla, 2011). For example, niche models were suitable for explaining the distribution mechanisms in a *Pinus tabulaeformis* forest, but the neutral model failed to explain any vegetation layer, though it was suitable for explaining either northern broad‐leaved or conifer and broad‐leaved mixed forest communities (Gao, Bi, & Yan, 2011). Other research suggests the random process of the neutral model at different successional stage of conifer and broad‐leaved mixed forest communities is the main ecological driver determining their SADs patterns at moderate and large sampling scales, and that SADs patterns can vary over growing seasons for the herb layer, for which neutral models performed better than niche models in the broad‐leaved *Korean pine* mixed communities of north temperate forests of the Changbai Mountains (Yan, Zhang, & Zhao, 2012; Zhang et al., 2015). Accordingly, every vegetation community may be located at some point along this continuum based on the relative contributions of niche and neutral processes to its composition (Bar‐Massada et al., 2014).

The great challenge remains the difficulty of measuring neutrality in real communities when addressing niche–neutrality questions (Bar‐Massada et al., 2014). The obvious differences among distinctive species in ecological habits, complex topography, competitive exclusion, niche differentiation, and succession processes might all cause that the potential specificity of certain patterns was covered. By using a modeling approach, we showed that patterns of SADs were scale‐dependent, suggesting that SADs at different scales were likely structured by different ecological processes. The neutral process strongly affected the generation of diversity patterns in China's subtropical secondary forests, but how the relative contribution of niche processes impacted community assembly could be denied. The problem of how the moments of the SADs scale is complex and likely related to that of the species–area relationship. However, as explored in this paper, analyses and models of the SADs can be useful in practical applications and provide insight into the assembly of ecological communities.

## AUTHOR CONTRIBUTIONS

Idea and study design: Anchi Wu, Xiangwen Deng; Sample plot survey: Anchi Wu, Xiangwen Deng, Yiran Jing, Wenhua Xiang, Shuai Ouyang, Wende Yan, Xi Fang; Statistical analysis: Anchi Wu, Xiangwen Deng, Honglin He, Xiaoli Ren; Manuscript writing: Anchi Wu; Discussion and revision: Anchi Wu, Xiangwen Deng, Honglin He, Wenhua Xiang. All authors have read and approved the content of the manuscript.

## Data Availability

We have uploaded the data along with the revised manuscript. We want to choose to use the Dryad data repository.
